# Analysis of protein-protein docking decoys using interaction fingerprints: application to the reconstruction of CaM-ligand complexes

**DOI:** 10.1186/1471-2105-11-236

**Published:** 2010-05-11

**Authors:** Nobuyuki Uchikoga, Takatsugu Hirokawa

**Affiliations:** 1 Japan Biological Informatics Consortium, 2-45 Aomi, Koto-ku, Tokyo, Japan; 2 Computational Biology Research Center, Advanced Industrial Science and Technology, 2-42 Aomi, Koto-ku, Tokyo, Japan

## Abstract

**Background:**

Protein-protein docking for proteins with large conformational changes was analyzed by using interaction fingerprints, one of the scales for measuring similarities among complex structures, utilized especially for searching near-native protein-ligand or protein-protein complex structures. Here, we have proposed a combined method for analyzing protein-protein docking by taking large conformational changes into consideration. This combined method consists of ensemble soft docking with multiple protein structures, refinement of complexes, and cluster analysis using interaction fingerprints and energy profiles.

**Results:**

To test for the applicability of this combined method, various CaM-ligand complexes were reconstructed from the NMR structures of unbound CaM. For the purpose of reconstruction, we used three known CaM-ligands, namely, the CaM-binding peptides of cyclic nucleotide gateway (CNG), CaM kinase kinase (CaMKK) and the plasma membrane Ca^2+ ^ATPase pump (PMCA), and thirty-one structurally diverse CaM conformations. For each ligand, 62000 CaM-ligand complexes were generated in the docking step and the relationship between their energy profiles and structural similarities to the native complex were analyzed using interaction fingerprint and RMSD. Near-native clusters were obtained in the case of CNG and CaMKK.

**Conclusions:**

The interaction fingerprint method discriminated near-native structures better than the RMSD method in cluster analysis. We showed that a combined method that includes the interaction fingerprint is very useful for protein-protein docking analysis of certain cases.

## Background

Protein-protein interactions are important events for regulating biological functions. A network of protein-protein interactions is considered scale-free type, rather than random [1-3], suggesting that from this network of protein-protein interactions we can find proteins interacting with various targets. Such proteins, referred to as hub proteins, are intrinsically disordered protein [1,4,5], which is one of the problems associated with predicting protein complexes from the rigid-body docking approach because of protein flexibility. In general, the rigid-body docking process is composed of four different steps: selection or building of molecular structures, docking for generating complex structures, refinement of models and finally the scoring or ranking step. We adopted the ensemble docking approach for the rigid-body docking analysis after taking protein flexibility into consideration, because backbone flexibility, resulting from the large conformational changes in the protein structure, is one of the important factors of protein-protein interactions [6].

A selection step is generally used to define conformational space in structure analysis. There are different methods for generating multiple structures: molecular dynamics (MD), normal-mode analysis (NMA), and 3-dimensional (3D) structural data generated by nuclear magnetic resonance (NMR). These methods are often combined to account for backbone and side-chain flexibilities [7-10]. However, for rigid-body docking with large conformational changes, backbone flexibility should be considered before side-chain rearrangement. Multiple structures associated with rigid-body docking could provide the information required for taking the backbone flexibility into account in ensemble docking analysis [11]. Following this step, side-chain flexibility could be induced locally at the docking sites of the receptor and ligand complex using the soft-docking method of modifying the side-chain volumes [12,13].

**Figure 1:**
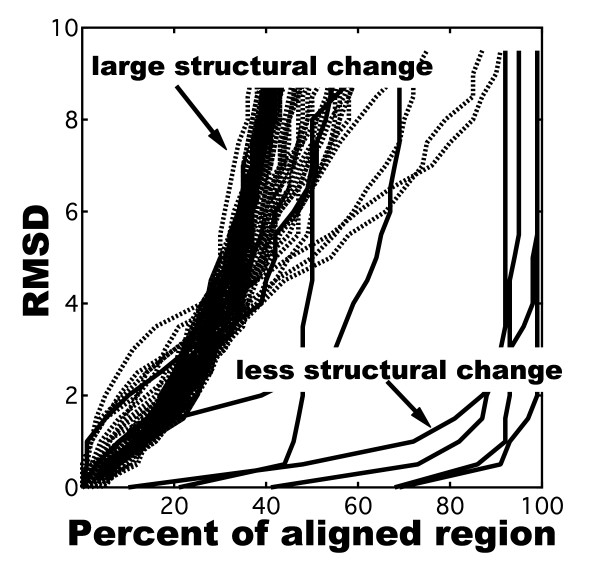
**Structure differences between the bound and unbound forms in protein-protein interaction using GDT plots**. Dotted lines represent comparison of the NMR-derived structures (1DMO) of 30 unbound states of CaM with the CaM structure extracted from the CaM-CNG complex (1SY9). Rigid lines show comparison between the bound and unbound structures of CAPRI targets: T09, 10, 14, 18-21.

Ensemble docking analysis can generate various complex structures based on the rigid-body data, and usually contains many false-positive structures. Therefore, another post-docking step could be added to the ensemble docking process to search for near-native structures. Cluster analysis is one such reliable approach available for use in this post-docking process. In this case, it is necessary to use certain parameters that would specify similarity or distance between the complex structures. There can also be issues in comparing structures using the root mean square distance (RMSD). Global Distance Test (GDT) plot [14] is a 2D diagram of the similarity of two molecular conformations based on searching sequence alignment regions with less RMSD, which is used for evaluating predicted structures in protein structure prediction contest, CASP (the Critical Assessment of protein Structure Prediction). Figure 1 is a GDT plot showing the differences in structure between the bound and unbound state of docking targets. As shown in Figure 1, there are two types of conformational differences between the unbound and bound forms in protein-protein interaction. One type shows less structural changes, which is typical of docking targets. The others diverge even when small portions are aligned - in this particular case they are calmodulin (CaM) and some of the critical assessment of prediction interactions (CAPRI) targets. It is, therefore, an important and a challenging problem to be able to predict the protein-protein interaction of a protein with large conformational changes. To tackle the problem of docking in proteins with large conformational changes, the RMSD value, although still useful, often depends on the superposition step. For comparing the structures of complexes generated from the docking process, we therefore introduced a protein interaction profile, called interaction fingerprints (IFPs), which is composed of binary states of interacting amino acid residues, as a scale for measuring unique similarities between the complex structures.

IFP is useful for classifying or selecting representative complex structures, and has been previously used for analyzing the protein-ligand docking problems rather than analyzing the protein-protein interactions [15,16]. IFP allows examination of the properties of protein-protein interactions simply by comparing the docking structures in terms of their interaction patterns, for example by using the Tanimoto index (Tc-IFP) [15,16]. It is, therefore, possible that by using IFP one could select the near-native structures at the contact residue string level, rather than obtaining the exact complex structure at the Cartesian coordinate level.

In the present study, we used an ensemble of native (bound state) and 30 different NMR-derived structures of calmodulin (CaM), which largely differed from the native structure as indicated by the dotted lines in Figure 1, for our analysis. We then applied IFP to the protein-protein post-docking process for the reconstruction of various CaM-ligand complexes. IFPs were obtained from the generated complex structures and were used for the cluster analysis. Subsequently, we analyzed relationship between the energy profile and structural similarity or distance using Tc-IFP or RMSD in order to improve reliability of the overall process for proteins with large conformational changes. We also briefly discussed about searching near-native groups by cluster analysis in terms of their energy scores.

## Results

**Figure 2:**
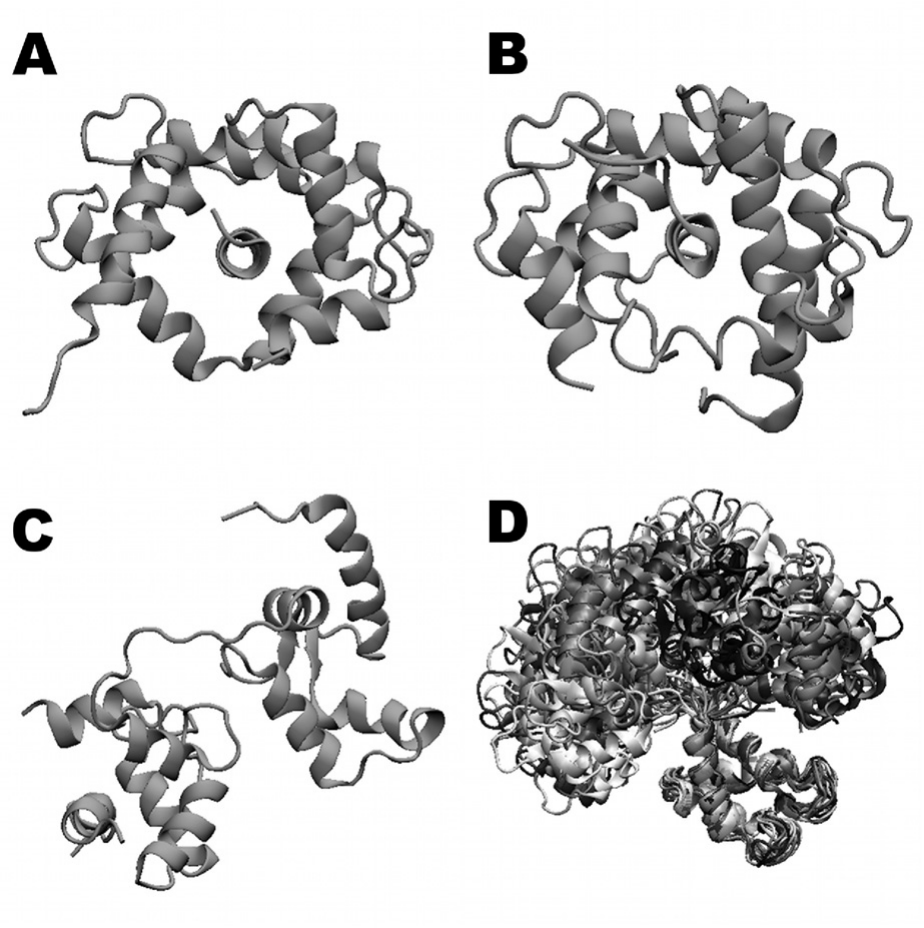
**Structures of ligand-bound and unbound CaM**. Three different types of CaM complexes were used in this work: complexes with A) CNG, B) CaMKK and C) PMCA. (D) Thirty structures of the single unbound state CaM are shown after superposing of the N-terminal domains.

Decoy structures were obtained from the rigid-body docking simulations using one of the 3 peptides (CNG, CaMKK, or PMCA) as a ligand and CaM multi-structures generated from NMR analysis (Figure 2). Through one docking simulation round for each CaM-ligand pair, we obtained 2000 decoys. For each peptide, two types of decoys, 60000 decoys called as "a-decoys" and 2000 decoys called as "b-decoys", were generated from 30 apo-CaMs and one bound state CaM, respectively. Some complex structures were rejected in the process of side-chain rearrangement because of their unavailable arrangements, as detailed in the "Protein docking process" of the Methods section. Consequently, out of 62000 (31 x 2000) decoys for each peptide, the numbers of decoys used for post-docking analysis were 61830 for PMCA, 61909 for CaMKK and 61854 for CNG.

### Interaction energy scores of decoys

**Figure 3:**
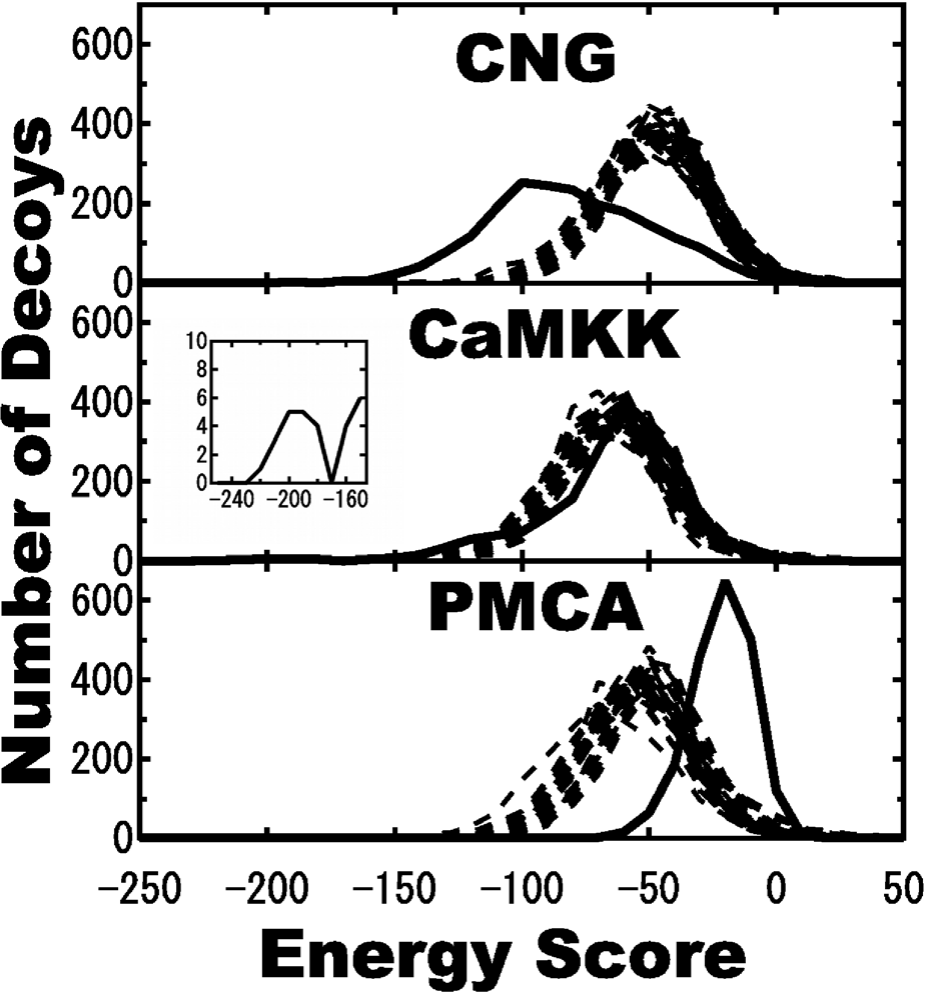
**Distributions of interaction energy scores **. Dotted and rigid lines indicate a-decoys and b-decoys, respectively. Mean values and standard deviations of a-decoys for CN, CaMKK and PMCA were -43.0 ± 21.6 (n = 59,854), -60.2 ± 23.0 (n = 59,909), and -48.6 ± 21.89 (n = 59,830), respectively. Mean values and standard deviations of b-decoys for CNG, CaMKK and PMCA were -76.13 ± 31.84 (n = 2,000), -60.23 ± 28.69 (n = 2,000), and -16.52 ± 12.06 (n = 2,000), respectively. The inset in the CaMKK panel shows the presence of one peak for b-decoys around -200.

Figure 3 shows the distribution of interaction energy scores in decoys of CaM-peptide ligand complexes. Here, we plotted a-decoys and b-decoys separately, and adopted ZRANK score as the interaction energy score (see the Methods). As shown, for each ligand, a-decoys have similar peaks and deviations, whereas the distributions of the b-decoys were different from those of the a-decoys.

In the case of CNG, the b-decoy set showed lower mean values and larger standard deviations than the a-decoys. In the case of CaMKK, distributions of the b-decoys were similar to those of the a-decoys. However, the b-decoys showed larger deviations because of the existence of a low energy peak around -200 in the interaction energy score (inset of CaMKK panel), which was completely divided at -170 and included 18 b-decoys. In contrast to CNG, PMCA had more b-decoys with higher interaction energy scores than the a-decoys.

### Structural similarities between the decoys and the native structure

**Figure 4:**
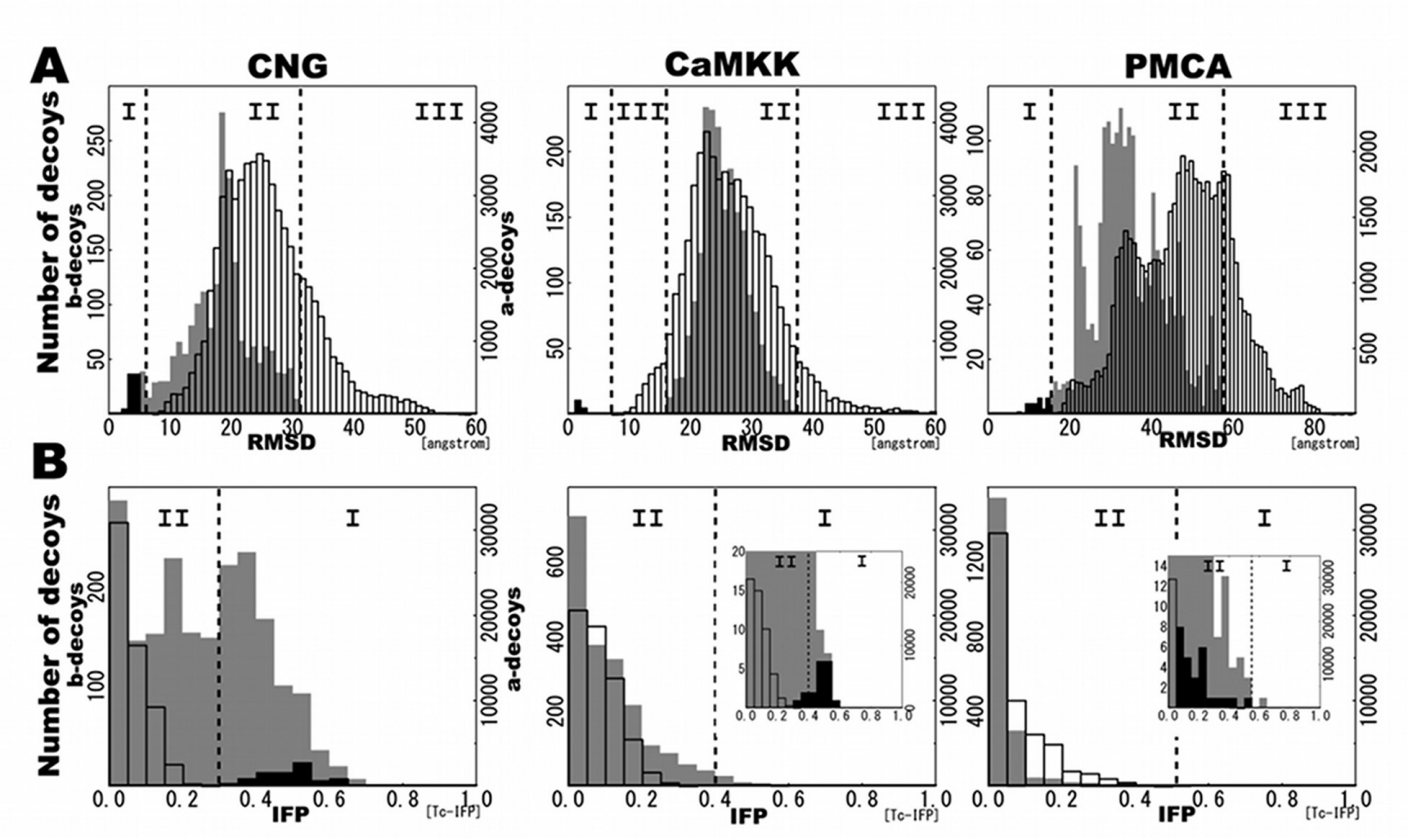
**Distributions of similarities between the decoys and native structures**. Decoys were obtained using (A) RMSD and (B) Tc-IFP. Open bars indicate a-decoys (right vertical axis). Gray bars indicate b-decoys (left vertical axis). Solid bars indicate decoys with less than 5.0 angstrom RMSD for CNG and CaMKK, and less than 10.0 angstrom RMSD for PMCA.

In order to analyze the structural landscape of decoys, it is important to investigate whether the near-native decoys could be discriminated from all decoys. Thus, we next investigated which method, Tc-IFP or RMSD, is better able to discriminate the near-native decoys from all decoys. Figure 4 shows distributions of a-decoys (open bar), near-native b-decoys with RMSD less than 5.0 angstrom (solid bar), and other b-decoys (gray bar). Areas I, II, and III indicate areas containing only b-decoys, overlap of b-decoys and a-decoys, and only a-decoys, respectively. As shown in Figure 4A, the b-decoys are distributed in the lower RMSD compared to the a-decoys, which are distributed in bell-shaped manner in the case of CNG and CaMKK. There were 133 and 1867 of CNG b-decoys, and 18 and 1982 CaMKK b-decoys in areas I and II, respectively. B-decoys in area I of CaMKK have the lowest energy groups of around -200 energy scores, as previously seen in the inset of Figure 3, which could be divided from a-decoys. In area I of CNG, b-decoys have energy scores between -50 and -150.

In the case of Tc-IFP (Figure 4B), most a-decoys in all three cases were distributed around 0.0, indicating that almost all a-decoys did not have any similarity with the native interactions. On the other hand, b-decoys were distributed more broadly than a-decoys in the case of CNG and CaMKK. We found 660 and 1340 CNG b-decoys, and 51 and 1949 CaMKK b-decoys in the Tc-IFP areas I and II, respectively, indicating that the b-decoys contained more near-native structures than the a-decoys. B-decoys in the Tc-IFP area I of CNG were consisted of decoys with RMSD lower than 5.0 angstrom and other b-decoys. The latter b-decoys have slightly higher energy scores than the former decoys. In CaMKK, b-decoys in area I consisted of portions of b-decoys with RMSD lower than 5.0 angstrom and other b-decoys with energy scores between -100 and -150. Comparison of the Tc-IFP and RMSD results indicate that for both CNG and CaMKK, the Tc-IFP method produced more b-decoys in area I than the RMSD method. Furthermore, the b-decoys, obtained using the Tc-IFP method, could be better divided into groups than the RMSD method. Thus, it appears that the Tc-IFP is a more desirable method than RMSD for discriminating b-decoys from other decoys.

In contrast to the CNG and CaMKK b-decoys, the number of PMCA b-decoys in areas I and II, obtained using the RMSD method, were 46 and 1954, respectively, and those obtained using the Tc-IFP method were 1 and 1875, respectively. Clearly, the Tc-IFP method resulted less number of PMCA b-decoys in area I than the CNG and CaMKK b-decoys. This might have resulted from the high-energy scores of b-decoys (Figure 3) and the smaller interacting surface of the CaM-PMCA complex than the respective CNG and CaMKK complexes, as shown in Figure 2.

To examine the difference between the Tc-IFP and RMSD, we also paid attention to the b-decoys with low RMSD. Three types of distributions were found in Tc-IFP: (1) only area I type, all 79 b-decoys with low RMSD in CNG are found in area I; (2) almost area I type, 3 out of 18 b-decoys with low RMSD in CaMKK are in area II; and (3) only area II type, all 27 b-decoys with low RMSD in PMCA are in area II. Thus, in the case of globular cognate structure of CaM (i.e., CaM-CNG and CaM-CaMKK complexes), b-decoys with low RMSD values were found in an area of Tc-IFP that scored higher similarity than the a-decoys.

### Cluster analysis

**Figure 5:**
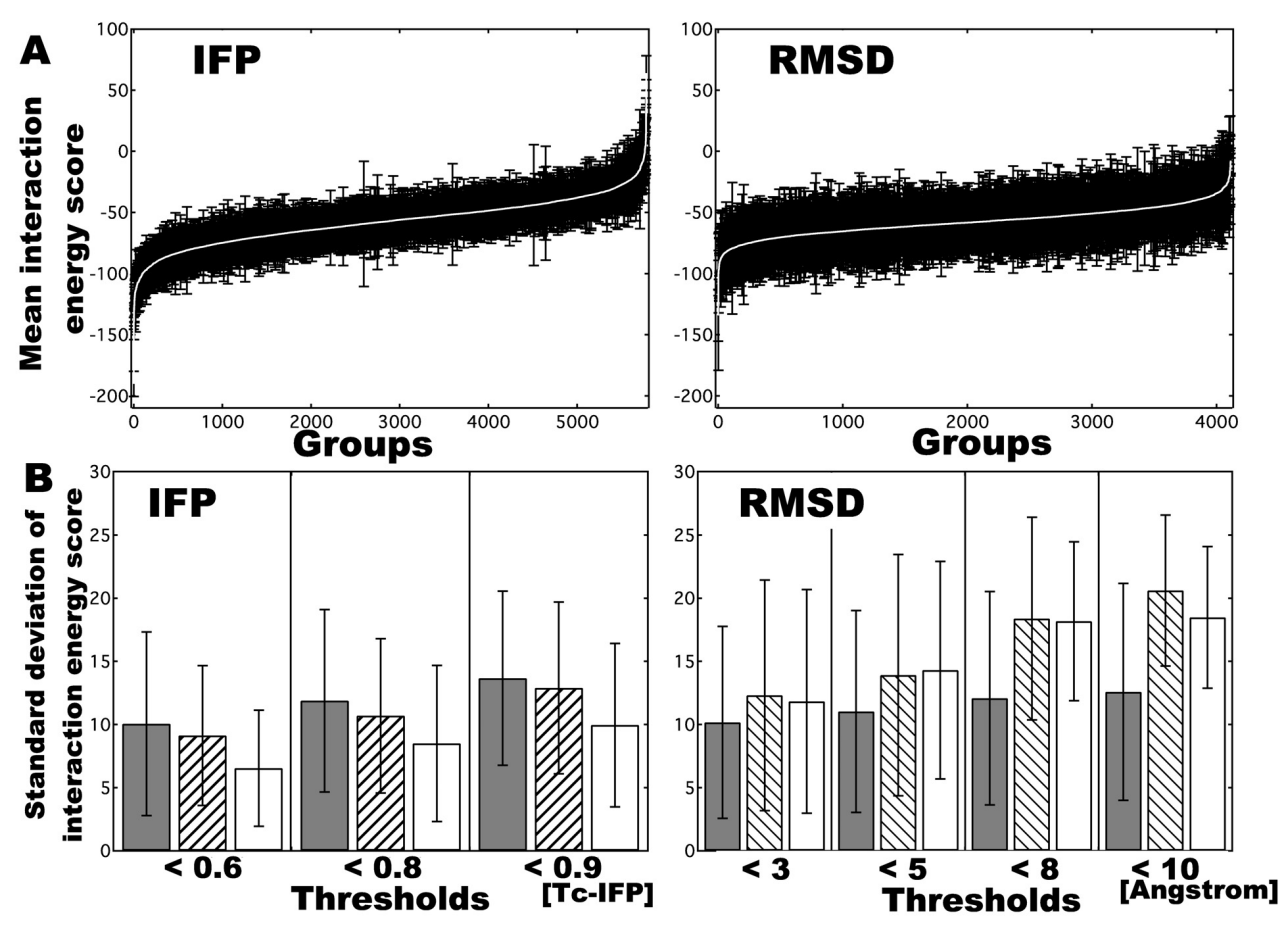
**Deviations of interaction energy scores**. A: Example of interaction energy scores for each divided group with error bars (n = 61909) generated from CaMKK decoys. In the left panel (IFP), energy score is derived from 0.8 threshold of Tc-IFP, and in the right panel (RMSD), energy score is derived from 8 angstroms of RMSD. B: Interaction energy score deviations for each threshold: for Tc-IFP, the thresholds are 0.6, 0.8 and 0.9; for RMSD, the thresholds are 3, 5, 8 and 10 angstroms. Detail values are indicated in the text. Gray, striped and open bars indicate CNG, CaMKK, and PMCA, respectively.

Cluster analysis is useful for choosing near-native decoys without prior knowledge of native structures. Using a certain threshold, decoys could be classified into groups composed of similar decoys. Uniformity of every group could then be investigated by using interaction energy scores. When groups with more uniformity of interaction energy scores were obtained, the used parameters were considered to be better suited for the cluster analysis. We, therefore, used the unweighted pair group method with arithmetic mean (UPGMA) to construct hierarchical tree from the results obtained by Tc-IFP and RMSD analyses. After generating a dendrogram, groups of decoys obtained using Tc-IFP or RMSD were divided according to their similarities. Figure 5A shows two examples of deviations of interaction energy scores of groups of decoys generated from CaMKK; results from the Tc-IFP analysis is derived from 0.8 threshold and results from the RMSD analysis is derived from 8.0 angstroms threshold. In this case, result obtained from the Tc-IFP analysis appears to be better than that from the RMSD analysis because magnitudes of the error bars are smaller in the Tc-IFP than in the RMSD. Next, we used 0.6, 0.8 and 0.9 as thresholds of Tc-IFP and 3.0, 5.0, 8.0 and 10.0 angstroms as thresholds of RMSD, respectively. Figure 5B shows the standard deviations of interaction energy scores for every group with error bars; clearly, for each parameter, the deviations become large as the threshold was increased. In the case of CNG, deviations and error bars were almost of similar magnitudes irrespective of whether Tc-IFP or RMSD was used for the analysis. On the other hand, in the cases of CaMKK and PMCA, deviations and error bars obtained using Tc-IFP were of smaller magnitudes than those obtained using RMSD. These results suggest that in terms of interaction energy scores we could obtain more uniform groups by using Tc-IFP than by RMSD.

## Discussion

We introduced IFP for describing interaction profiles by taking protein flexibility into consideration. In the case of the globular cognate structure of CaM (i.e., CaM-CNG and CaM-CaMKK complexes), b-decoys scored higher similarities to the native complex than the a-decoys derived by the Tc-IFP analysis (Figure 4B). We also used the cluster analysis to determine the abilities of Tc-IFP and RMSD in dividing the decoys into groups of similar decoys according to the Tc-IFP or RMSD threshold. We obtained more uniform groups in terms of interaction energy scores by Tc-IFP than by RMSD (Figure 5). These results indicated that IFP can be used as one of the profiles of decoys for determining similarities among themselves in terms of interacting amino acids.

**Figure 6:**
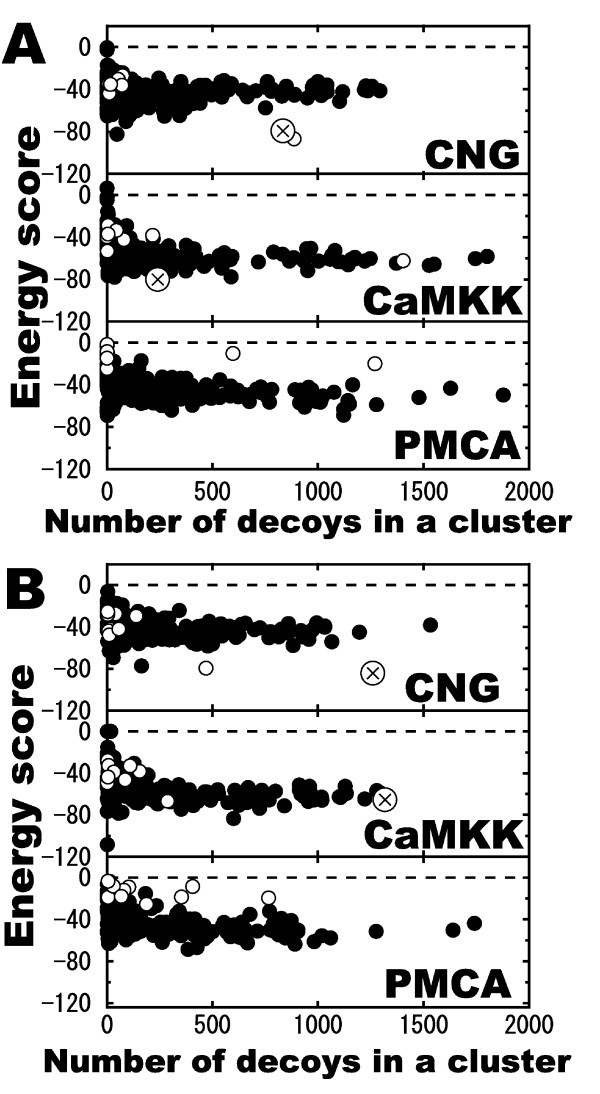
**Dispersion diagrams showing relationship between the number of decoys and energy scores in clusters**. Groups were obtained using (A) Tc-IFP and (B) RMSD. Energy scores and the numbers of decoys for each group are plotted. For each CaM structure, decoys were divided into 10 groups. Closed circles indicate groups consisting of only a-decoy. Open circles indicate groups consisting of only b-decoy. Open circles with a cross mark inside indicate near-native groups. In the case of PMCA, near-native groups were not found.

We then tried to discriminate the near-native clusters without the knowledge of native complex structure. For this purpose, decoys were classified into groups of similar decoys by cluster analysis, which was slightly different from the cluster analysis described in the Results section. In this analysis, 2000 decoys, derived from the structural data of each CaM-ligand pair, were divided into 10 groups by using the statistical computing "R" software. Because we used 31 CaM structures, we therefore had 310 (= 10 × 31) groups for each CaM-ligand complex. Accordingly, using both IFP and RMSD, we found near-native clusters (shown as open circles with a cross mark inside in Figure 6), having most number of interacting residues identical to the native complex or having highest similarities (S_frac_) to the native complex, for each ligand except for PMCA. In the case of CNG, we could discriminate the near-native groups in both Tc-IFP and RMSD by searching for groups with lower energy scores. The number of decoys in the near-native group was the second largest and the largest among the b-decoy groups obtained by Tc-IFP and RMSD analyses, respectively. Similarly, in the case of CaMKK, the near-native groups obtained by Tc-IFP analysis could be discriminated as a group with the lowest energy score among all groups, which includes the set of b-decoys with the lowest energy scores (shown in the inset of Figure 3). However, the number of decoys in the near-native groups was not so large. On the other hand, for CaMKK, the near-native group obtained by RMSD analysis consisted of the most number of b-decoys, although the energy score was lowest only among the b-decoy groups.

**Figure 7:**
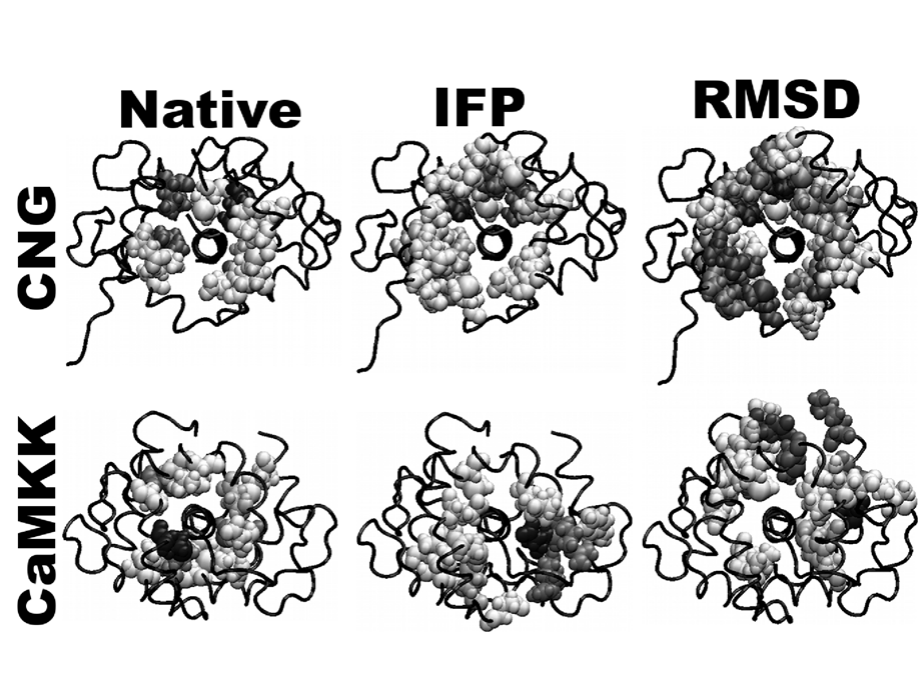
**Binding sites of near-native clusters in complexes of CaM with CNG (top panel) and CaMKK (bottom panel)**. Binding sites are depicted as van der Walls (VDW) balls. Darker VDW balls indicate the decoy residues interacting more with the ligand. Interaction clusters obtained using IFP are more similar to the native interactions than those obtained using RMSD.

Based on the energy scores we could discriminate the near-native groups in the case of CaM-CNG both by Tc-IFP and RMSD. However, in the case of CaM-CaMKK, energy score could not be the index for searching the near-native groups by RMSD, indicating that the RMSD method is unable to classify the decoys with the lowest energy scores (shown in Figure. 3). We, therefore, compared the interaction frequencies of near-native clusters. As shown in Figure 7, more frequently interacting residues were found on the surface of CaM in the near-native clusters obtained by RMSD than those found in the native structure and also than those in the near-native clusters obtained by IFP. This result suggests that by using the IFP method one could identify interactions similar to the native interactions. Therefore, the cluster analysis in combination with IFP, rather than with RMSD, appears to be a much better approach in predicting the near-native decoys without any prior structural knowledge.

In the case of the CaM-CaMKK complex, we obtained a set of desired near-native decoys with lowest interaction energy (Figure 3) and high Tc-IFPs (Figure 4). These results are explained by the structure of the CaM-CaMKK complex, which is different from those of the CaM-CNG and CaM-PMCA complexes. The globular cognate structure of CaM in the CaM-CaMKK complex differed only slightly from that of the CaM-CNG complex, indicating that the cavity of the CaM-CaMKK complex is narrower than that of the CaM-CNG complex, as shown in Figure 2. In contrast to the simple alpha helical structure of the CNG and PMCA ligand peptides, the CaMKK ligand peptide has an additional loop region in the C-terminal end. These structural features of the ligand have contributed in obtaining a set of near-native decoys for the CaM-CaMKK complex. In context with the shape of the ligand peptide, it is noteworthy that we found CaM-CNG b-decoys in which the CNG was bound to the CaM cavity in an inverse manner as compared to the native complex; this inverse binding of CNG might have contributed to the broad distribution of the CaM-CNG b-decoys (Figure 4B). In any general docking problem, any ligand with simple alpha helix-like pseudo-symmetrical shape might have similar binding characteristic of CNG.

In the case of CaM-PMCA, near-native decoys could be discriminated clearly from the native complexes using RMSD. On the other hand, in the case of Tc-IFP, less number of near-native decoys (Figure 4) and none of the near-native groups (Figure 6) were obtained for the CaM-PMCA complex. This is in contrast to the CaM-CNG and CaM-CaMKK complexes, in both of which the number of interacting residues are similar [17,18]. PMCA interacts with CaM using about half the number residues than those involved in CaM-CNG or CaM-CaMKK interactions [19], because it interacts only with the C-terminal lobe of CaM. In a general docking problem, this type of interaction is normally involved in "solvent-accessible ligand" and "open and shallow form of receptor" cases. That the number of near-native decoys for the CaM-PMCA complex was found to be less by our analysis, could be related to the fact that the decoys were obtained not only from the extended cognate structure of CaM (b-decoys), but also from some unbound states of CaM (a-decoys). Therefore, in order to obtain better near-native decoys following the post-docking process, we need to further improve on the IFP method in terms of physicochemical approach, and also need to develop a combination method of IFP and RMSD.

Although we could discriminate near-native decoys in CaM-CNG and CaMKK, diversity of decoys were also obtained using this method. To further develop the IFP method for solving the docking problem of proteins with large conformational changing, we need to generate better ensemble of bound structures by using MD or NMA methods for the cross-docking analysis.

## Conclusions

In the present study, IFP was introduced for describing interaction profiles, which could be used for comparing different structures of decoys by taking protein flexibility into consideration. In cluster analysis, IFP could clearly discriminate the near-native structures better than RMSD in terms of energy scores. We also showed that a combined method that included IFP was useful for protein-protein docking analysis. We believe that this method, in combination with other docking software, such as RosettaDock software that optimizes both side-chains and backbones, could be applied to other protein complexes deposited in the protein structure databases.

## Methods

To perform ensemble docking simulations, we used structural data of multiple forms of unbound states and a single rigid ligand-bound state of a receptor protein, which in this case is calmodulin (CaM). Trimming and optimization of side-chains were taken into consideration in soft docking for obtaining IFPs. IFP, in combination with cluster analysis, was used to classify the near-native structures of the CaM complexes by comparing their structures with different known CaM structures.

### Protein structural data

Multiple structural data of a receptor protein were used for ensemble docking simulations. In the present study, we used CaM as the receptor protein. CaM is a widely studied protein that undergoes large conformational changes both in the unbound and bound states, and has been normally used in molecular biological studies rather than in docking problems. Structurally, CaM consists of two globular domains at the N- and C-termini connected by a central helix domain, known as a dumbbell-like shape. Because of the presence of a disordered region in the central domain, a single CaM molecule can structurally switch between the open and closed conformational states. Indeed, structure analysis using NMR revealed that a single CaM molecule existed in a variety of structural conformations [20]. Such structural changes allow CaM to bind with various target proteins, and the bound states are classified into open and closed conformations. In the present study, we used the interacting parts of the cyclic nucleotide gateway (CNG), CaM kinase kinase (CaMKK) and the plasma membrane Ca^2+ ^ATPase pump (PMCA), as CaM ligands, all of which were shown to contain alpha helix-rich structures. Figure 2 shows the structures of three bound states of CaM-ligand complexes and a single unbound state of CaM. In their CaM-bound states, the CNG (Figure 2A) [17] and CaMKK (Figure 2B) [18] interacted with the bended form of CaM, but in the opposite directions. In contrast, PMCA bound to the C-terminal part of the extended form of CaM (Figure 2C) [19]. For each ligand, 31 different structures of CaM were used as receptor molecules for the rigid-body docking simulations. Structures of thirty unbound states of CaM (Figure 2D) were obtained from the Protein Data Bank (PDB; PDBID: 1DMO, containing 30 NMR structures) [20]. The other CaM structures used in this study were the bound state structural data of 'Model 1' of chain 'A' in 1SY9 (20 NMR structures), 1CKK (30 NMR structures), and 1CFF (26 NMR structures); these structural data corresponded to the bound states of CaM with the respective ligands used in the present work. For all structures, the amino acid sequence of the CaM protein, which is 148 amino acids long, was same except for an asparagine residue at position 127 in the unbound form was an aspartic acid residue in the bound form.

Structures of ligands used in this study were chosen from the bound state structural data: CaM bound to CNG olfactory channel (PDBID: 1SY9, Model 1 of chain B), CaM bound to CaMKK (PDBID: 1CKK, Model of chain B) and CaM bound to PMCA (PDBIB: 1CFF, Model 1 of chain B). The three chosen ligands were short peptides derived from CNG, CaMKK and PMCA and contained 20, 26 and 19 amino acid residues, respectively.

### Protein docking process and cluster analysis

In this work, we considered backbone flexibility by using rigid-body ensemble docking with multiple structures derived from NMR analysis. Side-chains of amino acids in decoys were optimized through side-chain trimming process, which is similar to the induced fit process.

To obtain decoys by rigid-body docking, we used ZDOCK ver.2.3 [21] with the option for sampling at 6-degree rotational steps. Using Fast Fourier Transform, ZDOCK searches for all possible binding orientations of a ligand along the surface of a receptor protein, optimizing desolvation, shape complementarity and electrostatics [21]. The top 2000 structures, along with their ZDOCK scores, were used as candidates of near-native structures. The docking score was calculated by considering several interaction properties, e.g., shape complementarity, desolvation, and electrostatics [21,22]. For side-chain rearrangement, volume of each side-chain of the receptor protein was modified to that of alanine before docking. The coordinates of alanine side-chains were modelled by using the software MOE ver. 2007.09. After rigid-body docking, structures of top 2000 relative positions of a ligand bound to a receptor structure were obtained. Side-chains of the protein-ligand complex structures were next optimized by using SCWRL3 [23], followed by substituting the trimmed side-chain containing receptor structural data with those of the original structural data. This side-chain rearrangement process is similar to induced fit analysis. We did not optimize the backbones because multiple NMR generated CaM structures corresponded to backbone flexibility; moreover, CaM structure is almost rigid except for a central helical domain connecting the N- and C-terminal globular domains. During this process, we found some decoys with unavailable arrangement side-chain, which was defined as the arrangement that took more than 10 min of CPU time for optimization. Such decoys were not be used in the post-docking process.

We call the protein-ligand complex structures, generated through this docking process, as "decoys." For full-atom complex structures modelled by MOE ver. 2007.09, we used ZRANK [24] to determine the interaction energy scores, which is a linear weighted sum of the van der Walls, electrostatic and desolvation energies.

We also performed cluster analysis using distance matrices. For generating tree of decoys based on similarities, we used the unweighted pair group method with arithmetic mean (UPGMA) algorithm, which is one of the pair group methods and is often used for generating phylogenetic tree of life. We used the statistical computing R software ver.2.8.0 for this analysis.

### Comparison of decoys with interaction fingerprint (IFP)

RMSD is generally considered a suitable and powerful parameter when comparing structures of molecular complexes. However, in the case of proteins with large conformational changes (Figure 1), it is sufficient to compare the interacting fragments rather than the whole structures [15,16]. For this purpose, profiles of interacting amino acid residue pairs were obtained by the dimplot command of the LIGPLOT program [25]. LIGPLOT counts the numbers of both hydrogen bond and non-bonded contact. In this work, we reconstructed CaM complexes, whose key interactions involve in hydrophobic residues [17-19]. Actually, the number of non-bonded contacts in the native CaM complexes are much higher than the hydrogen-bonded contacts; the number of non-bonded and hydrogen-bonded contacts are, respectively, 139 and 6 in CaM-CNG, 139 and 6 in CaM-CaMKK, and 57 and 0 in CaM-PMCA. In our docking process, we do not have any process involved in optimization of hydrogen bond. Therefore, we used the non-hydrogen bond contacts to generate the interaction profiles. Information on residue pairs were entered into a bit sequence, in which one bit corresponded to a residue pair. If a pair was found, the bit was assigned a numerical value of 1; if not, the bit was assigned a numerical value of 0.

After generating an interaction profile of molecular complexes, cluster analysis was performed. Similarity between the decoys and native molecular complexes was calculated as the Tversky similarity [26] as follows:

where *a *and *b *are the number of bits including queries *a *and *b*, respectively, and *c *is the common bit-number between *a *and *b*, *c *= *a *∩ *b*.

Parameters *α *and *β *varied from 1 to 0 independently. When *α *= 1 and *β *= 1, the similarity between the queries *a *and *b *could be calculated as follows:

indicating similarity between the queries *a *and *b*, which is known as the Tanimoto index (Tc-IFP). We used Tc-IFP when comparing decoys to native interactions. When *α *= 1 and *β *= 0, the ratio of the common bit-number to query *a *as IFP of native was calculated as follows:

This equation includes the bit-number of query *b *implicitly, through index *c*. *S*_*frac *_is the ratio of native amino acid pairs included in a decoy to all the native pair numbers. The numbers of bits in the native structures are invariable, which are 25 bits in CNG, 57 in CaMKK, and 17 in PMCA. Therefore, values of index *S*_*frac *_are discontinuous. We used index *S*_*frac *_to compare decoys with native structures because when query *b *has many more bits than query *a*, the value of index *S*_*Tanimoto *_loses linearity with the bit number of query *b *[27]. IFPs were subsequently used to perform cluster analysis to compare interactions of decoys, which are independent of the methods used for the superposition of 3D structural data. The index of *S*_*Tanimoto *_was used to quickly calculate whole pairs of decoys.

## List of abbreviations

IFP: interaction fingerprints; Tc-IFP: Tanimoto index; RMSD: root mean square distance; CaM: calmodulin; CNG: cyclic nucleotide gateway; CaMKK: CaM kinase kinase; PMCA: plasma membrane Ca^2+ ^ATPase pump; UPGMA: unweighted pair group method with arithmetic mean

## Authors' contributions

TH conceived the docking and post-docking process. NU performed the whole process and analyzed the data. NU drafted the manuscript and prepared the figures. All authors discussed the results, provided critical comments and approved the final manuscript.
